# Intoxication by Cyanide in Pregnant Sows: Prenatal and Postnatal Evaluation

**DOI:** 10.1155/2015/407654

**Published:** 2015-05-26

**Authors:** André T. Gotardo, Isis M. Hueza, Helena Manzano, Viviane M. Maruo, Paulo C. Maiorka, Silvana L. Górniak

**Affiliations:** ^1^Research Center of Veterinary Toxicology (CEPTOX), Department of Pathology, School of Veterinary Medicine and Animal Science, University of São Paulo, 13635-900 Pirassununga, SP, Brazil; ^2^Institute of Environmental, Chemical and Pharmaceutical Sciences, Federal University of São Paulo (ICAQF-UNIFESP), Campus Diadema, 09913-030 Diadema, SP, Brazil; ^3^College of Veterinary Medicine and Animal Science, Federal University of Tocantins, BR 153, Rural Zone Km 112, 77804-970 Araguaina, TO, Brazil

## Abstract

Cyanide is a ubiquitous chemical in the environment and has been associated with many intoxication episodes; however, little is known about its potentially toxic effects on development. The aim of this study was to evaluate the effects of maternal exposure to potassium cyanide (KCN) during pregnancy on both sows and their offspring. Twenty-four pregnant sows were allocated into four groups that orally received different doses of KCN (0.0, 2.0, 4.0, and 6.0 mg/kg of body weight) from day 21 of pregnancy to term. The KCN-treated sows showed histological lesions in the CNS, thyroid follicle enlargement, thyroid epithelial thickening, colloid reabsorption changes, and vacuolar degeneration of the renal tubular epithelium. Sows treated with 4.0 mg/kg KCN showed an increase in the number of dead piglets at birth. Weaned piglets from all KCN-treated groups showed histological lesions in the thyroid glands with features similar to those found in their mothers. The exposure of pregnant sows to cyanide thus caused toxic effects in both mothers and piglets. We suggest that swine can serve as a useful animal model to assess the neurological, goitrogenic, and reproductive effects of cyanide toxicosis.

## 1. Introduction

Cyanide is a ubiquitous chemical in the environment and has been associated with many intoxication episodes in human and animals. Cyanide can be released by industrial processes including metal processing, electroplating, and plastic and chemical synthesis [1–3]. Considering only United States, it is produced 300,000 t of this substance, a year, to feed their industries of electroplating, paper, and plastic and in the extraction of gold [4]. This ion is also present in tobacco smoke [5], and smoke inhalation is a common cause of cyanide poisoning during fires [6]. Some drugs of medicinal importance, such as laetrile and nitroprusside can release cyanide [7]. Moreover, many plants for human and animal feed, such as* Manihot *sp. (cassava),* Linum *sp.,* Lotus *sp.,* Phaseolus lunatus*, and* Sorghum *sp., contain cyanogenic glycosides that release cyanide. The concentration of this substance can be as high as 100–800 mg/kg of plant material [3, 8].

The mechanism of acute cyanide intoxication is well known; it occurs through the inactivation of mitochondrial cytochrome c and disruption of aerobic respiration, which results in potentially fatal cellular hypoxia and cytotoxic anoxia [9]. However, given the constant presence of cyanide in the environment, the most important adverse effects of this compound are currently assumed to be due to chronic toxicity. Thus, there is a need to carefully assess the potential toxic effects from prolonged exposure to cyanide.

Indeed, neuropathies have been attributed to prolonged exposure to low concentrations of cyanide. In humans, chronic and spontaneous degenerative diseases, such as spastic paraparesis or “konzo” [10] and tropical ataxic neuropathy [11, 12], have been associated with high cassava consumption. Furthermore, prolonged cyanide exposure has been associated with Parkinson's disease and cognitive impairment [13, 14].

Tobacco smoking and cassava consumption have been implicated in the pathogenesis of ocular diseases such as tobacco-alcohol amblyopia [15, 16], retrobulbar neuropathy of pernicious anemia [17], Leber's hereditary optic neuropathy [18, 19], West Indian amblyopia [20, 21], Jamaican optic neuropathy [22], tropical amblyopia [23], and Cuban optic neuropathy [24].

In addition, pancreatic and tropical diabetes in humans and animals have been associated with the ingestion of cyanogenic plants [25, 26], especially cassava [27].

Experimental studies in dogs, goats, and rats have shown that chronic cyanide exposure causes impaired body growth, goiter, and lesions of the central nervous system (CNS) [28–30]. In addition, we have verified that the long-term administration of cyanide to growing pigs promotes neurotoxic, hepatotoxic, and nephrotoxic effects [31].

Although studies have been conducted on chronic cyanide intoxication in various animal species, little is known about its potentially toxic effects on development. Fetal malformations have been linked to maternal consumption of cyanogenic plants in animals such as pigs, horses, sheep, and cattle [32–36] as well as in humans [37, 38]. Moreover, studies of goats conducted in our lab have shown that maternal cyanide exposure is associated with fetal abortion and retrognathia [39]. We verified these findings in studies with rats, where pups from dams treated with different doses of cyanide and/or thiocyanate (the active metabolite resulting from the biotransformation of cyanide) exhibited similar toxic effects, some of which were observed only at weaning [40].

We have previously demonstrated that the pig is a useful animal model for assessing chronic cyanide toxicity [31], and others have found it suitable for reproductive toxicology studies [41, 42]. The aim of this study was to evaluate the toxic effects, in both dams and their offspring, of maternal exposure to potassium cyanide (KCN) during pregnancy in pigs.

## 2. Materials and Methods

This study was conducted at the University of São Paulo (USP) Experimental Station, Pirassununga, SP, Brazil (S21°58′, W47°27′). The procedures were approved by the USP Animal Ethics Committee, and all animal care and handling was performed by experienced personnel under veterinary supervision.

### 2.1. Animals, Feeding, and Experimental Design

Landrace-Large White sows, 240 days old, were bred to one male pig of the same breed. All females were negative for antibodies to Aujeszky's disease and for infections with* Campylobacter *sp.,* Mycobacterium avium*,* Brucella suis*, and* Leptospira *sp.

The day of breeding was defined as gestational day 1 (GD1), and pregnancies were confirmed by ultrasound (US) apparatus (Scanner 100 Vet, Pie Medical) on GD21. After this confirmation, females began to receive the experimental rations. Pregnant sows were randomly allocated into four treatment group (*N* = 6 per group at the beginning of the experiment) and administered varying doses of KCN (Merck, Germany), in mg per kg of body weight (mg/kg BW), as follows: 0 (KCN0 group), 2.0 (KCN2 group), 4.0 (KCN4 group), and 6.0 (KCN6 group).

The purpose of this study was to assess for embryotoxic effects of cyanide using swine as a model organism. Given that embryo implantation in swine starts on day 13 after breeding and ends on day 18 [43], KCN was administered from GD21 to parturition (approximately day 114 of pregnancy) twice a day, with one half dose administered between 7:00 and 07:30 and the other half dose administered between 17:30 and 18:00, mixed with 10 g of starch biscuits in individual troughs. The control group received only the biscuits. Water and food were freely available. During gestation, the dams were weighed weekly.

### 2.2. Biochemical and Hormonal Evaluations

Blood samples were collected via jugular venipuncture using heparinized syringes at the 21st, 50th, 80th, and 110th days of pregnancy. Blood serum was frozen and stored at −10°C until analysis. Commercial kits (CELM, Brazil) were used for the determination of albumin, glucose, urea, creatinine, aspartate aminotransferase (AST), and gamma glutamyltranspeptidase (GGT) levels using automatic biochemical system (SBA-200 CELM). The plasmatic concentrations of thyroxine (T_4_) and triiodothyronine (T_3_) were measured on GD110 using commercial radioimmunoassay kits (Coat-A-Count Siemens).

### 2.3. Ultrasonographic Evaluation and Pathological Study in Sows

Ultrasonographic evaluation was performed on GD45, GD55, and GD65 in each pregnant swine to measure the following fetal parameters: crown-rump length (CRL), thoracic diameter (TD), abdominal diameter (AD), and biparietal diameter (BPD).

On GD110, one pregnant sow from each group was euthanized; the amniotic fluid was collected for chemical analysis. Fragments of the CNS, thyroid glands, lungs, pancreas, liver, and kidneys were collected and fixed in 10% buffered formalin, embedded in paraffin, cut into 5 *μ*m sections, and stained by hematoxylin and eosin (HE).

### 2.4. Evaluations in Piglets

Immediately at birth, each neonate's body weight and gender were recorded. Each newborn was then examined carefully for gross abnormalities [44]. Two newborn pigs, a male and female from each mother, were then euthanized to collect fragments of the CNS, thyroid glands, lungs, pancreas, liver, and kidneys. From birth (PND1) to three months of age (PND120), each piglet was weighed weekly and the weight gains were calculated. The absolute weights and weight gains of the piglets were analyzed separately by gender. Blood samples from each piglet were taken via jugular venipuncture immediately after birth and on the 7th, 14th, 21st, and 45th days of life for the evaluation of the same biochemical parameters assessed in their mothers.

At the end of the experimental period (PND120), the piglets were euthanized. Fragments of the CNS, thyroid, pancreas, liver, and kidney were collected and fixed in 10% buffered formalin, embedded in paraffin, cut into 5 *μ*m sections, and stained by hematoxylin and eosin (HE).

### 2.5. Analytical Procedures

Thiocyanate levels of gilts were dosed in serum at GD21, GD50, GD80, and GD110, in amniotic fluid on GD110 and into colostrum immediately after parturition by spectrophotometry using the method outlined by Pettigrew and Fell [15], with modifications specific to our study. Briefly, 0.2 mL of the biological materials were added to 1.8 mL of a 10% trichloroacetic acid solution and centrifuged at 10,000 rpm for 20 min. Next, 0.5 mL of supernatant aliquots was acidified with 0.25 mL of 1 M HCl. The samples were mixed after adding each of the following reagents: 50 *μ*L of saturated bromine water, 100 *μ*L of trioxide arsenious (20 g/L in 0.1 N NaOH), 0.9 mL of pyridine (10 mL of 12 N HCl, 60 mL of pyridine, and 40 mL of deionized water), and p-PDA reagent (2 g in 1 L of 0.5 M HCl; 3 : 1, prepared immediately before use). The reddish-pink complex that formed was read at 540 nm after 15 min in a spectrophotometer (Micronal B382 Micronal, S.A. Brazil) against a blank sample. Thiocyanate levels in the samples were read based on standard curves prepared with thiocyanate of known concentrations (25–300 *μ*mol/L). Data were expressed as *μ*Lmol/L.

The linearity of the analytical procedure was tested at concentrations of 25, 50, 75, 100, 200, and 300 *μ*mol/L of thiocyanate in 10 samples for each concentration. The coefficient of determination was 0.9911, slope 0.0008097, and the *y*-axis intercept in 0.005267. The intralaboratory precision, expressed by the coefficient of variation intra- and interday was obtained by analyzing the levels of thiocyanate in standard samples of 100 *μ*mol/L in water, and pool samples of serum, colostrum, and amniotic fluid, processed five times each on two different days. The coefficient of variation ranged from 1.3 to 5.8% intra- and 2.1 to 4.8% interday. The thiocyanate recovery was from 95.5 to 98.3 for serum; 94.5 to 96.6 for colostrum; and 98.8 to 98.2 for amniotic fluid added to the standard solution of thiocyanate at concentrations of 100 and 200 *μ*mol/L, respectively.

### 2.6. Statistical Analysis

Weight gain data, serum biochemistry, ultrasound measurements, and serum thiocyanate levels were analyzed using a mixed linear model (Proc Mixed) for each treatment. The animals were nested within the treatments, and repeated measurements of the variables were taken over time. The animals were considered a random factor in the model.

Hormone serum levels, birth weights, and thiocyanate levels in the colostrum were analyzed statistically by one-way analysis of variance (ANOVA) followed by Dunnett's test. The frequency of dead piglets was analyzed using Fisher's exact test, with each treatment compared to the controls.

Data are reported as the means ± SEM and were analyzed using SAS software (Version 9.2; SAS Institute, Cary, NC). In all cases, the probability of significant differences was set at *α* = 0.05.

Calibration and concentration curves of thiocyanate in the different biological fluids versus the time, taken after treatments, were made using the GraphPad Prism 5:00 (Graphpad 2007).

## 3. Results

Pregnant sows did not show clinical signs of acute cyanide intoxication during the experimental period. All the dams survived the treatment throughout the study. There was no significant difference between controls and KCN-treated sows in body weight gain (Table 1, *P* > 0.05).

Fetal deaths were diagnosed by ultrasonographic evaluation in three females from the KCN4 group and in one pregnant female from KCN6 group between 21 and 45 days of gestation. Females that failed pregnancy were withdrawn from the experiment and replaced with pregnant females.

**Figure 1:**
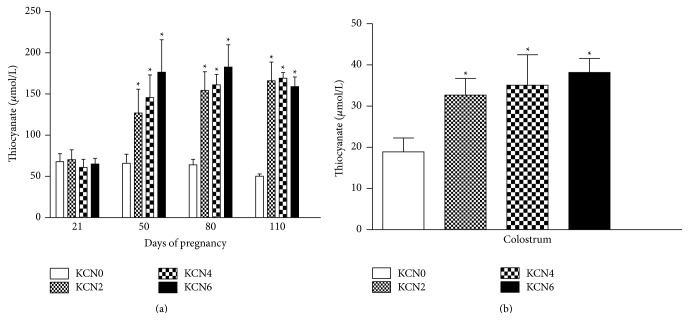
Thiocyanate levels (means ± S.E.M.) in plasma (a) and colostrum (b) of sows allocated into four treatment groups: KCN0, KCN2, KCN4, and KCN6 that received, respectively, 0.0, 2.0, 4.0, and 6.0 mg/kg of body weight of cyanide from day 21 of pregnancy to term. ∗*p* < 0.05 compared to control.

Biochemical analysis of serum collected during pregnancy showed fluctuations of multiple parameters, but these parameters remained within the normal ranges for all swine (data not shown). Similarly, no changes in T_3_ (control 0.039 ± 0.005, KCN2 0.033 ± 0.001, KCN4 0.034 ± 0.002, and KCN6 0.034 ± 0.002) and T_4_ (control 0.84 ± 0.4, KCN2 0.9 ± 0.3, KCN4 0.79 ± 0.1, and KCN6 0.93 ± 0.2) levels were found on the 110th day of gestation. On the other hand, plasma thiocyanate levels were increased on GD50, GD80, and GD110 in females from all treated groups (Figure 1, *P* < 0.05). Colostrum concentrations of thiocyanate were increased in all dams treated with cyanide (Figure 1, *P* < 0.05). Amniotic fluid evaluated from one female in each group showed increased thiocyanate levels in a dose-response fashion (KCN0 20.5 *μ*mol/L, KCN2 51.1 *μ*mol/L, KCN4 55.9 *μ*mol/L, and KCN6 65.7 *μ*mol/L).

Ultrasonographic evaluation at GD45, GD55, and GD65 demonstrated no significant changes in the morphometric measurements (CRL, TD, AD, and BPD) of the fetuses in each group (data not shown).

**Figure 2:**
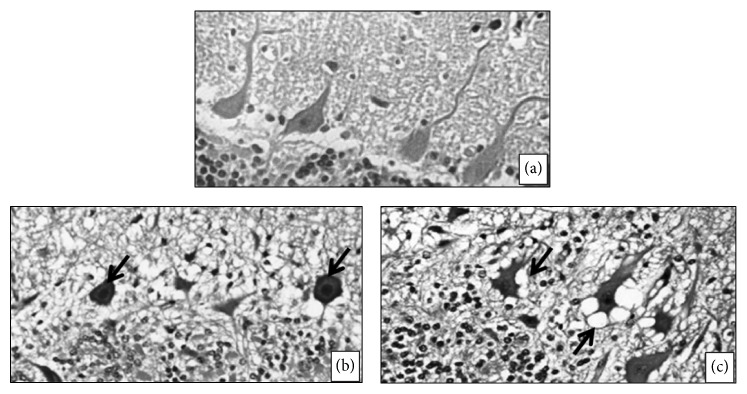
Light photomicrograph of pregnant sows CNS: (a) animal from control group; ((b) and (c), resp.) animals treated with 4.0 and 6.0 mg/kg of body weight of cyanide from day 21 of pregnancy to 110 term showing Purkinje cells with acidophilic cytoplasm (arrow; (b)) and vacuolar degeneration of Purkinje cells (arrow; (c)). Magnification: ×20, HE.

**Figure 3:**
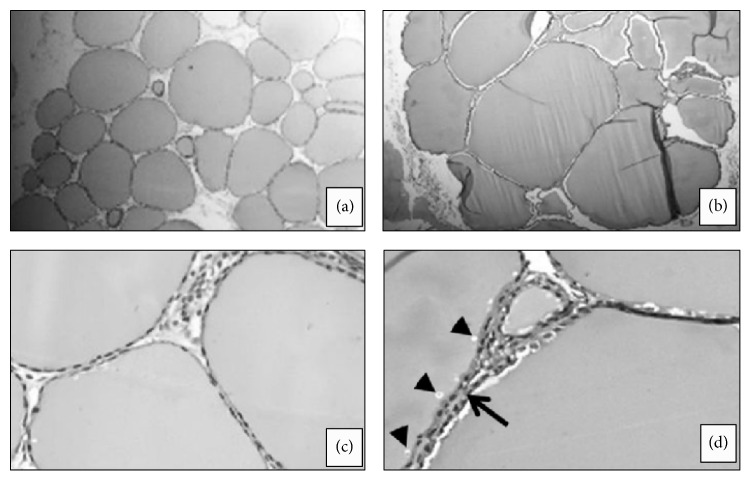
Light photomicrograph of pregnant sows thyroid: ((a) and (c)) animals from control group; ((b) and (d)) animals treated with 6.0 mg/kg of body weight of cyanide from day 21 of pregnancy to 110 term showing enlargement of thyroid follicles (b), thickening of the follicular epithelium (arrow; (d)), and reabsorption vacuoles in colloid (arrowhead; (d)). Magnification: ×4 ((a) and (b)); ×20 ((c) and (d)), HE.

**Figure 4:**
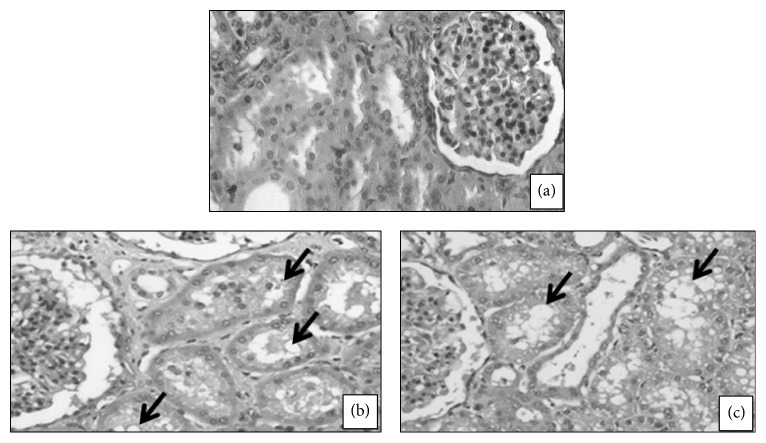
Light photomicrograph of pregnant sows kidney: (a) animal from control group; ((b) and (c), resp.) animals treated with 2.0 and 6.0 mg/kg of body weight of cyanide from day 21 of pregnancy to 110 term showing vacuolar degeneration of the renal tubular epithelium (arrow; (b) and (c)). Magnification: ×20, HE.

In histopathological assessments of euthanized pregnant sows at GD110, a greater number of Purkinje cells with acidophilic cytoplasm were found in all experimental sows, whereas vacuolar degeneration of Purkinje cells was observed only in sows from the KCN6 group (Figure 2). Compared to the control sows, the experimental sows had thyroid follicle enlargement, thickening of the cubic follicular epithelium and reabsorption vacuoles in colloid (Figure 3). Additionally, there was vacuolar degeneration of the renal tubular epithelium in sows from all groups exposed to KCN (Figure 4).

There was a significant increase (*P* = 0.002) in the number of dead piglets in the KCN4 group compared with the KCN0 group (Table 1). The body weight of the piglets at birth was unaffected (Table 1, *P* > 0.05) by the treatment.

The biochemical analysis of serum collected from piglets showed fluctuations of the different parameters, but these parameters remained within the normal ranges for swine (data not shown). At PND120, there was no treatment effect on the overall weight gain of male or female piglets (Table 1). No histopathological lesions were observed in euthanized newborns. At PND120, piglets in all experimental groups had similar histopathological changes as their mothers, including thyroid follicular enlargement and epithelial thickening and vacuolization (Figure 3).

## 4. Discussion

The administration of KCN orally was chosen aiming to mimic the main route of natural exposure to this species. The doses of KCN used here were chosen based on data from an earlier study conducted in our laboratory on male growing-finishing swine [31], in which the animals showed toxic effects of chronic exposure to cyanide but did not manifest any clinical signs of acute toxicity (e.g., dyspnea and convulsive seizures). Similarly, none of the pregnant females in this study showed clinical symptoms of acute cyanide intoxication during the period of KCN administration.

Given that the bitter taste of cyanide could compromise food consumption during the study [45], starch biscuits known to be highly palatable to swine were chosen to borne the cyanide. This procedure ensured that all females ingested the target doses of cyanide quickly (usually in less than 2 min) in each administration. In a previous study of toxicokinetics in pregnant sows, we verified that, following oral exposure to KCN, serum thiocyanate levels increase by 3 hrs, peak after 6 hrs, and remain elevated for up to 21 hrs [46]. Thus, we assumed that the administration of cyanide twice daily would ensure high serum levels of thiocyanate steadily, independently of the phase of pregnancy.

Although some studies have found an association between the reduction of body weight and prolonged exposure to cyanide in humans and animals [47–49], we did not detect a decrease in this parameter in any of the pregnant sows exposed to KCN. This result corresponds with our previous work conducted in growing-finishing pigs [31]. Likewise, studies evaluating cyanide exposure during pregnancy in goats [39] and rats [40] have not found differences in weight gain between the control and experimental animal groups. In the same manner, Tewe and Maner [50] did not find weight changes in pregnant pigs fed cassava containing high levels of cyanide.

Although no biochemical changes were observed in pregnant females exposed to KCN, the histopathology performed in these sows revealed kidney and liver damage. Lesions similar to those observed in our study have previously been described in studies of other species chronically exposed to cyanide [30, 51, 52], as well as in pigs [31]. It remains to be determined if these lesions are a consequence of toxicity directly from cyanide, thiocyanate, or both.

Studies clearly show that the CNS is an important target in prolonged exposure to cyanide in both humans [53] and animals [54–56]. In humans, it has been described to result in several neuropathies, such as spastic paraparesis (also called “konzo”), Parkinson's disease, cognitive impairment, and ocular pathologies [11, 13, 14, 16, 24, 57].

The pig is an emerging model system for studying the human CNS [58]. In a previous study in our lab, where cyanide was administered to growing pigs during the same period evaluated here, we found Purkinje cell degeneration and loss of cerebellar white matter in all groups treated with KCN [31]. Similar lesions were found in this study in sows exposed to cyanide. These findings strengthen our assertion that pigs should be used as an animal model to better understand the mechanism(s) of neurotoxicity from cyanide and/or its main metabolite.

Tropical pancreatic diabetes has been associated with chronic cyanide exposure through consumption of cassava in man [25] and was reproduced in studies of dogs fed cassava [59]. However, studies administering cyanide to different animal species [29, 60], including pigs [31], were not able to reproduce these diabetogenic effects despite the animals having islet cells functionality similar to humans [61]. Given that pregnancy itself has a diabetogenic effect due to a decrease in insulin sensitivity of maternal tissues and resultant increase in insulin demand [62, 63], pregnant sows would theoretically be even more susceptible to develop diabetes upon cyanide exposure. Thus, we hypothesized that our study would have offered all the conditions needed to reproduce tropical pancreatic diabetes in pigs. However, we did not find any changes in the parameters chosen to evaluate the diabetogenic effects of cyanide (i.e., glucose levels and histopathology). As we have previously shown that pregnant rats exposed to cyanide have increased levels of glucose and pancreatic islet degeneration [40], pregnant rats may be a better animal model to assess the pancreotoxic effects of cyanide.

Studies have found a correlation between low doses of chronic cyanide intake with a higher incidence of abortion in mares [32], sheep [36], and goats [39]. Chronic cyanide intake in low doses from tobacco smoke has similarly been associated with spontaneous abortion in women [64], although it remains unclear if this finding is specifically due to cyanide rather than other toxic compounds found in cigarettes.

Higher rates of fetal death, stillbirth, assisted birth, and mummified piglets were found in sows exposed to cyanide in this study. The most common infectious causes of reproductive disease in Brazil (*Campylobacter *sp.,* Mycobacterium avium*,* Brucella suis*, and* Leptospira *sp.) were ruled out in these animals. Many causes could be posited to explain these finding, but it is plausible to consider KCN as the most likely one. We therefore suggest that the pig is a good animal model system for studying the role of cyanide and/or its main metabolite in smoking-related abortions in women. In our study, as we did not detect malformations in newborn pigs, it was not possible to conclude if ultrasound evaluation in pigs could be valuable for early detection of malformations “in utero.”

Amniocenteses in this study revealed high levels of thiocyanate in the amniotic fluid of pregnant sows. Dencker et al. [65] and Schwartz [66] show that anionic substances, such as the weak acid cyanuric acid, can accumulate in the embryo and fetus. This may be due to the pH gradient in maternal plasma, which is more acidic than the pH of fetal plasma. Studies have thus observed an “imprisonment” of cyanide in the fetal organism [67]. In addition, it is known that the enzyme rhodanese is found in the placenta and the fetus [68]. Thus, it is possible that the “arrested” cyanide can be transformed into its main metabolite, resulting in indirect thiocyanate toxicity to the fetus. This hypothesis is additionally supported by a study in which cyanide or thiocyanate was administered to pregnant rats, and both substances were found to promote toxic effects in the fetuses [40].

The protocols that have been used to assess developmental toxicity in previous studies have certain limitations [69]. One such limitation is the lack of postnatal monitoring of the development of the offspring, which can result in erroneous conclusions about the developmental toxicity of the substances under examination. In fact, de Sousa et al. [40] evaluated prenatal exposure to cyanide in rats and found that alterations in pups were detected only at weaning. Our study supports these findings, as we identified certain toxic effects of cyanide in the piglets only at weaning.

## 5. Conclusion

In conclusion, pregnant sows exposed to cyanide had toxic effects in both mothers and piglets. However, the toxic effects in the offspring were observed belatedly. Therefore, the data presented here reinforces the hypothesis that a complementary evaluation of the neonate after weaning is a necessary component of studies on developmental toxicity. In addition, we suggest that swine can serve as a useful animal model to study the neurological, goitrogenic, and reproductive toxicity of cyanide.

## Figures and Tables

**Table 1:** Reproductive parameters from sows treated with KCN from day 21 of pregnancy to term and the body weight of their piglets at birth until 120 days old.

Parameters	Control (*n* = 6)	KCN (mg/kg per day)		
		2.0 (*n* = 6)	4.0 (*n* = 6)	6.0 (*n* = 6)
Body weight gain (kg) during pregnancy^a^	69.6 ± 4.6	55.2 ± 5.1	49.8 ± 4.1	59.4 ± 8.2
Number of assisted births	0 (5)^b^	0 (5)	4 (5)	1 (5)
Live piglets	57	55	36	51
Male	27	30	13	24
Female	30	25	23	27
Number of dead piglets	6	4	23^*∗*^	10
Stillbirths	5	4	14	7
Mummified fetuses	1	0	9	3
Subsequent total born piglets (mean)	12.6	11.8	7.2	9.4
Piglets body weight at birth^a^	1.68 ± 0.04 (57)^c^	1.64 ± 0.03 (55)	1.71 ± 0.07 (36)	1.60 ± 0.05 (51)
Overall weight gain (kg) of piglets at 120 days				
Males	55.25 ± 3.82	52.47 ± 2.43	54.07 ± 2.97	52.79 ± 3.49
Females	53.82 ± 3.24	52.35 ± 2.78	51.62 ± 1.97	51.62 ± 2.99

^a^Mean ± S.E.M.

^b^Number of deliveries.

^c^Number of piglets weighed.

^*∗*^ *P* < 0.05 compared with controls.
